# A Global Transcriptomic Analysis Reveals Body Weight‐Specific Molecular Responses to Chronic Orange Juice Consumption in Healthy Individuals

**DOI:** 10.1002/mnfr.70299

**Published:** 2025-10-30

**Authors:** Layanne Nascimento Fraga, Dragan Milenkovic, Isabella de Araújo Esteves Duarte, Saivageethi Nuthikattu, Camille Perella Coutinho, Franco Maria Lajolo, Neuza Mariko Aymoto Hassimotto

**Affiliations:** ^1^ Food Research Center (FoRC) and School of Pharmaceutical Sciences University of São Paulo São Paulo Brazil; ^2^ Plants For Human Health Institute, Department of Food, Bioprocessing and Nutrition Sciences North Carolina State University Kannapolis North Carolina USA; ^3^ Division of Cardiovascular Medicine, Department of Internal Medicine University of California Davis California USA

**Keywords:** blood pressure, body mass index, flavanones, inflammation, lipid metabolism, orange juice, transcriptomic

## Abstract

Consumption of orange juice (OJ) has been linked to a lower incidence of cardiovascular diseases. However, molecular mechanisms underlying these health benefits remain unclear. This study aimed to investigate the molecular pathways involved in the health benefits of chronic OJ consumption through global transcriptomic approach in peripheral blood mononuclear cells (PBMCs). Healthy volunteers consumed 500 mL of OJ daily for 60 days. PBMCs were isolated from the blood, and the gene expression profiling was performed using Clariom microarrays. Genomic analysis revealed 1705 differentially expressed genes, including IL6, IL1β involved in inflammation, GSK3B, RIPK1 related to lipid metabolism, and NAMPT, NLRP3 involved in blood pressure regulation. Moreover, the expression of 66 miRNAs, 19 long‐non‐coding RNAs (lncRNAs), and 67 small nucleolar RNAs (snoRNAs) was modulated. Moreover, subgroup analysis revealed different transcriptomic responses between normal‐weight (NW) and overweight (OW) individuals. Genes related to lipid metabolism and adipogenesis were differentially expressed only in OW, including GSK3B, GRK6, miR‐548i, and miR‐1292‐3p. In contrast, genes related to the inflammation process, like STAT3, MAPK, and miRNAs (miR‐1185‐2‐5p), were observed only in NW. These findings suggest that body weight may influence molecular response to bioactive compounds in OJ and provide information for personalized recommendations on the consumption of flavonoid‐rich foods.

## Graphical abstract

This study aimed to investigate the molecular pathways involved in the health benefits of chronic orange juice (OJ) consumption through global transcriptomic approach in peripheral blood mononuclear cells (PBMCs). Healthy volunteers consumed 500 mL of OJ daily for 60 days. PBMCs were isolated from the blood, and the gene expression profiling was performed using Clariom microarrays. Genomic analysis revealed 1705 differentially expressed genes, including mRNAs, miRNAs, long‐non‐coding RNAs (lncRNAs), and small nucleolar RNAs (snoRNAs). Furthermore, subgroup analysis revealed different transcriptomic responses between normal‐weight and overweight individuals.

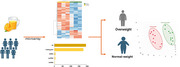

## 1 Introduction

Cardiometabolic diseases, such as hypertension, obesity, and diabetes, are the major causes of premature death in Brazil and worldwide [1]. High consumption of foods rich in fat, sugar, and sodium is associated with an increased occurrence of these comorbidities [2]. In contrast, a diet low in these components and high in fruits and vegetables has been linked to promoting better health [3]. Orange juice (OJ) is widely consumed in Brazil and is one of the primary sources of flavonoids in this population [4, 5]. The main classes of flavonoids include flavanones (hesperidin and naringenin) which have demonstrated antioxidant, anti‐inflammatory, and antiproliferative activities [6, 7, 8]. As a result, OJ consumption may be a strategy for improving cardiometabolic health [9, 10, 11, 12].

OJ and hesperidin induced changes in global gene expression, many of them implicated with adhesion, infiltration, and lipid transport [13], and blood pressure regulation [14, 15]. Genes of nicotinamide phosphoribosyltransferase (NAMPT) and pentraxin 3 (PTX3), found elevated in hypertensive patients, were downregulated after 12 weeks of OJ consumption by pre‐ and Stage 1‐hypertensive individuals [14]. These changes may be associated with reductions in blood pressure and body fat percentage in healthy individuals, previously observed in our studies, after chronic consumption of OJ, which was also linked to increased excretion of flavanone Phase II metabolites [11, 16]. Furthermore, citrus flavanone metabolites, hesperetin 7‐glucuronide and 3‐(4′‐hydroxyphenyl) propanoic acid, have been found to protect pancreatic β‐cells from oxidative stress, modulating the expression of proteins involved in insulin, glutathione metabolism, and inflammation (TNF‐α and NF‐κB pathways) signaling pathways [17]. These metabolites also significantly modulate protein expression in high‐glucose exposed cells, affecting proteins related to cell adhesion, inflammation, and endoplasmic reticulum protein processing pathways [8].

Despite promising results regarding the benefits of OJ consumption, the underlying mechanisms of action and considerable variability in response remain poorly understood. Therefore, this study aimed to investigate the molecular mechanisms associated with chronic OJ consumption using a transcriptomic approach in peripheral blood mononuclear cells (PBMCs).

## 2 Material and Methods

### 2.1 Material

Pasteurized OJ (*Citrus sinensis* L. *Osbeck* var. Pera), obtained in June of 2019, was supplied by Fundecitrus (Araraquara, Brazil), located in southeastern São Paulo state at 23°23′19″ S and 48°43′22″ W. The juice was poured into 1 L flasks and immediately stored at −20°C. The details regarding the chemical composition of juice were previously described [11].

### 2.2 Study Design

The complete study design was previously described by Fraga et al. [11]. Among the 85 volunteers enrolled in this study, 20 volunteers (10 men and 10 women) were selected for the presented nutrigenomic study through transcriptomic analysis. The 20 healthy volunteers of both sexes and aged between 21 and 36 years, with no evidence of chronic disease, were included in the final analysis. Exclusion criteria were as follows: cardiovascular, gastrointestinal, hepatic, or renal diseases; diabetic; alcohol consumption; vegetarianism; use of vitamin and mineral supplements, antibiotics, antacids, or medications for diarrhea or constipation; smoking; aversion to OJ; and pregnant, breastfeeding mothers or those taking hormone therapy for menopause.

The study was conducted in accordance with the Declaration of Helsinki, and all procedures were approved by the Ethics Committee of the School of Pharmaceutical Science‐University of São Paulo, São Paulo (CAAE 14344819.0.0000.0067) and the Ethics Committee of the University Hospital of São Paulo (CAAE 14344819.0.3001.0076) and were registered on the Brazilian Registry of Clinical Trials at https://ensaiosclinicos.gov.br/rg/RBR‐8pgbfpv (UTN code U1111‐1257‐7414). Written informed consent was obtained before the commencement of the study. Clinical trial settings and data collection were performed at the clinical unit of the University Hospital, University of São Paulo, Brazil.

Three days before the intervention, volunteers were instructed to restrict their consumption of citrus (orange, lemon, and grapefruit) and their derivatives, as well as strawberries, passion fruit, coffee, chocolate, wine, and teas. After the wash‐out period, each subject consumed 500 mL of OJ daily over 60 days. At home, volunteers were instructed to divide the total daily dose into two equal portions and to store the OJ in a refrigerator. On the first (T0) and last day (T60) of the intervention, blood samples were collected after an overnight fasting period (12 h). Blood pressure and anthropometric parameters were assessed, and a 24 h‐dietary recall (R24h) was performed. During the protocol period, the volunteers were contacted by phone, twice a week, to ensure adherence to the protocol; they were also asked to maintain their lifestyle and usual dietary habits, only avoiding citrus‐containing foods.

### 2.3 Isolation of Human Peripheral Blood Mononuclear Cells (PBMC)

Human PBMCs were isolated from 8 mL of whole blood collected in BD Vacutainer CPT Mononuclear Cell Preparation Tube with Sodium Heparin, according to the manufacturer's specifications. After washing with phosphate‐buffered saline (PBS), a cell count was performed, and the pellet was stored in a freezer at −80°C for later analysis.

### 2.4 Total RNA Isolation and Microarray Analyses

The RNA was extracted from the PBMCs using the miRNeasy Mini Kit according to the manufacturer's specifications (Qiagen, Hilden, Germany). The RNA quality was checked by electrophoresis in a 1% agarose gel, whereas the quantity was assessed by measuring absorbance at 260 and 280 nm using a NanoDrop ND‐1000 spectrophotometer (Thermo Scientific, Wilmington, DE, USA).

For transcriptomics analysis, we used Clariom D array for humans, containing over 6 million probes for protein‐coding genes but also protein non‐coding genes such as miRNAs, long‐non‐coding RNAs (lncRNAs), and small nucleolar RNAs (snoRNAs) (Thermo Fisher Scientific, Santa Clara, CA, USA). RNA (100 ng) was used to prepare cRNA and sscDNA using Thermo Fisher Scientific GeneChip WT PLUS reagent Kit. SscDNA, amount of 5.5 𝛍g, was fragmented by uracil‐DNA glycosylase (UDG) and apurinic/apyrimidinic endonuclease 1 (APE 1) and labeled by terminal deoxynucleotidyl transferase (TdT) using the DNA Labeling Reagent that is covalently linked to biotin. Fragmented and labeled ssCDNA samples were used for hybridization, staining, and scanning by using Thermo Fisher Scientific WT array hybridization protocol following the manufacturer's protocol. Hybridization of fragmented and labeled ssCDNA samples was done using GeneChipHybridization oven and the arrays were washed then stained using GeneChip Fluidics Station. The arrays were scanned using GeneChip Scanner 3000 7G (Thermo Fisher Scientific). Quality control of the microarrays and data analysis were performed using Thermo Fisher Scientific Transcriptome Analysis Console software version 4.0.2. Pair‐wise comparisons between biological conditions were applied using specific contrasts. Probes with FDR‐adjusted *p* < 0.05 were considered to be differentially expressed between conditions.

### 2.5 Bioinformatic Analysis

Pathway‐enrichment analyses were conducted using the bioinformatic tool GeneTrail 3, version 3.2 (https://genetrail.bioinf.uni‐sb.de/), as a platform to access Kyoto Encyclopedia of Genes and Genomes (KEGG), and Wikipathways databases, Biocarta, and applying the following stings: over‐presentation analysis; null hypothesis (for *p* value computation)‐ two‐sided; method to adjust *p* values‐Benjamini–Hochberg; significance level −0.05. STRING software version 11.0 (https://string‐db.org/) was used for protein–protein interaction analyses, including physical and functional associations. Analysis of potential transcription factors that regulate the differentially expressed protein‐coding genes was conducted using the platform Enrichr (https://maayanlab.cloud/Enrichr/).

For each miRNA identified as differentially expressed, we performed target analysis. miRNA genes targets were predicts using the bioinformatic tool Mienturnet (http://userver.bio.uniroma1.it/apps/mienturnet/), using the data available from the miRTarBase. For each lncRNA identified as differentially expressed, we performed target analysis. lncRNA gene targets were predicted using the bioinformatic tool LncRRIsearch, considering the energy threshold −16 kcal/mol (http://rtools.cbrc.jp/LncRRIsearch/).

**FIGURE 1:**
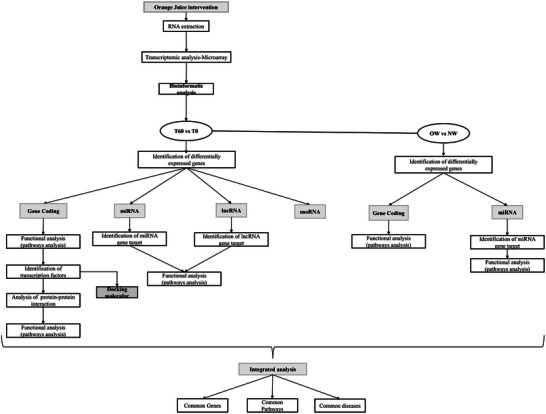
Flowchart of the step‐by‐step of bioinformatic analysis.

The association of OJ flavanone metabolites modulate protein‐coding genes with human diseases was conducted using the Comparative Toxicogenomics Database, *p* value < 0.05 (http://ctdbase.org/). The integrative and comparative analysis among the three groups was conducted using the software Biovenn (https://www.biovenn.nl/index.php) (Figure 1).

### 2.6 Molecular Docking

Molecular docking was performed using the Swiss Dock docking analysis tool (http://www.swissdock.ch/docking) [18, 19], between the transcription factors (nuclear factor kappa B subunit 1 [NFKB], aryl hydrocarbon receptor [AHR], peroxisome proliferator‐activated receptor‐α [PPARA], activating transcription factor 4 [ATF4], plasminogen activator, urokinase [PLAU], nuclear respiratory factor 1 [NRF1], proto‐oncogene [MYC], Yin‐Yang 1 [YY1], ETS transcription factor ELK4 [ELK4], fator de transcrição p65 [RELA], retinoic acid receptor RXR‐alpha [RXRA], interferon regulatory factor 9 [IRF9], and tumor protein 52 [TP53], and the Phase II flavanone metabolites (hesperetin 3‐glucuronide, hesperetin 7‐glucuronide, hesperetin 3‐sulfate, naringenin 4‐glucuronide, and naringenin 7‐glucuronide), and flavanone catabolites (3‐(3′‐hydroxy‐4′‐methoxyphenyl) hydracrylic acid; 3‐(3′‐hydroxy‐4′‐methoxyphenyl) propionic acid). Chemical structures of the compounds were obtained from the PubChem database (https://pubchem.ncbi.nlm.nih.gov/). The protein 3D structure was obtained from Uniprot Data Bank (https://www.uniprot.org/). Obtained 3D interactions were visualized using the Chimera program (https://www.rbvi.ucsf.edu/chimera) [20]. Interactions lower than −6 kcal/mol were considered significant.

## 3 Results

Previously in the same study, we reported that consumption of 500 mL of OJ/day for 60 days reduced blood pressure and body fat percentage [11]. In the present study, we aimed to identify potential underlying molecular mechanisms of action through global gene expression.

### 3.1 Orange Juice Consumption Modulates Global Gene Expression

**FIGURE 2:**
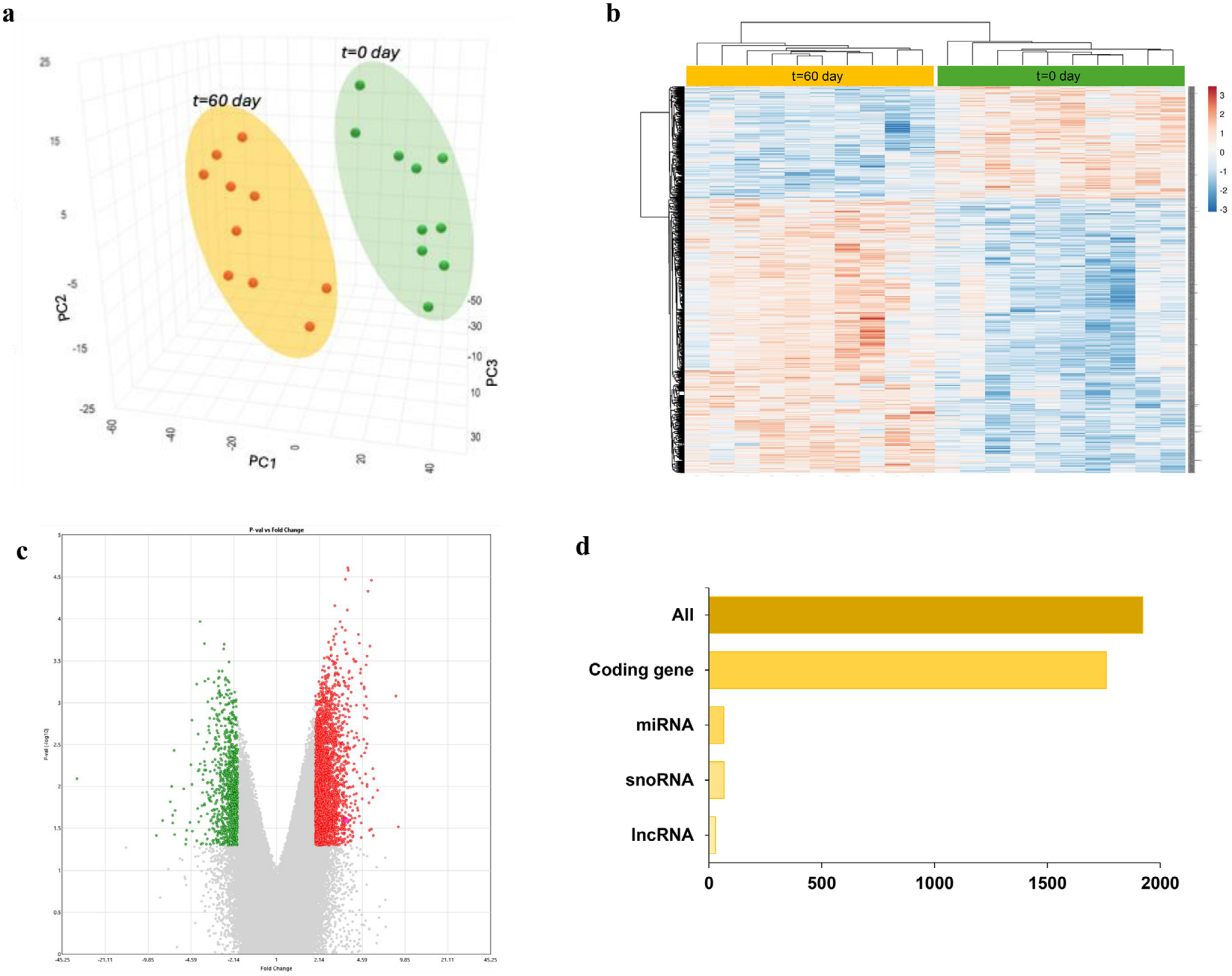
Modulation of global gene expression in peripheral blood mononuclear cells (PBMC) of humans after orange juice consumption (*n* = 20). (a) Partial least squares‐discriminant analysis (PLS‐DA) of gene global expression profile after 60 days of orange juice (OJ) consumption. (b) Heatmap obtained with hierarchical cluster analysis of global gene expression. (c) Volcano plot of global gene expression. (d) Several differentially expressed coding genes and non‐coding genes (miRNA, snoRNA, and lncRNA).

To assess the effect of OJ on the global gene expression in PBMCs, 20 volunteers out of 85 who completed the trial were included in the transcriptomic study. We first performed global gene expression profile comparisons by applying principal component analysis (PCA). As shown in the Figure 2A, the global gene expression profiles of volunteers after 60 days of regular consumption of OJ differ from global gene expression profile at the beginning of the study. This observation suggests that regular intake of OJ can impact global gene expression. Similarly, hierarchical clustering of the global gene expression profiles shows two groups, one corresponding to the global gene expression profiles at the beginning of the study and second grouping gene expression profiles of volunteers after 60 days of OJ consumption (Figure 2B).

Our next step was to perform statistical analysis of the gene expression data to identify genes having significant change in expression. Gene expression was compared after 60 days of OJ consumption versus at the beginning of the study. A total of 3790 oligonucleotides were differentially expressed, 2487 were downregulated (fold change from varying from −1.5 to −72) and 1303 were upregulated (fold change varying from 1.5 to 4.5) (Figure 2C). These oligonucleotides included 1705 coding genes (mRNA), 66 miRNA, 19 lncRNA, and 67 snoRNA (Figure 2D).

#### 3.1.1 Orange Juice Consumption Induces Changes in the Protein‐Coding Genes Expression

**FIGURE 3:**
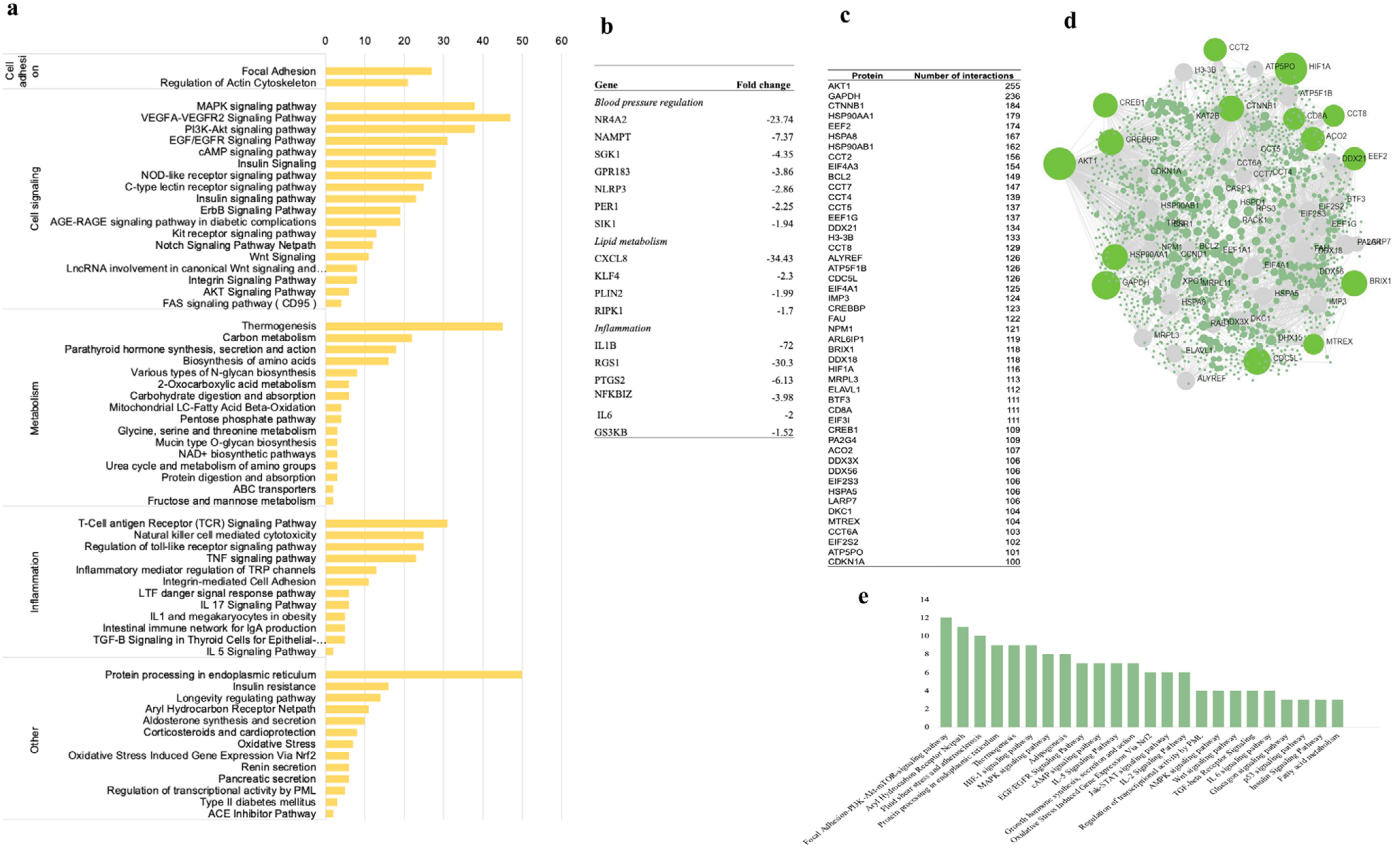
(a) Pathways analysis of genes differentially expressed after 60 days of orange juice (OJ) consumption. (b) List some genes differentially expressed and involved in blood pressure regulation, lipid metabolism, and inflammation pathways. (c) List of proteins with >100 predicted protein–protein interactions (STRING database). (d) Network of interactions between differentially expressed proteins. The size of highlighted nodes is related to the number of interactions; the largest nodes represent the largest number of interactions. (e) Pathways in which proteins with high interactions are involved.

Among the 1705 coding genes differentially expressed after consumption of OJ, 1673 were downregulated, and 32 were upregulated. To identify cellular functions in which these coding genes are involved in, an enrichment pathway analysis using the GeneTrial tool was performed. This analysis demonstrated that protein coding genes are involved in processes that can regulate blood pressure (aldosterone synthesis and secretion, renin secretion, and ACE inhibitor pathway); lipid metabolism (thermogenesis, adipogenesis, mitochondrial fatty acid beta‐oxidation, and others); inflammation (regulation of toll‐like receptor signaling pathway, TNF signaling pathway, IL 17 signaling pathway, and others); cell adhesion (focal adhesion, regulation of actin cytoskeleton); cell signaling (MAPK signaling pathway, VEGF‐VEGFR2 signaling pathway, PI3K‐Akt signaling pathway, EGF/EGFR signaling pathway, cAMP signaling pathway, insulin signaling, and AGE‐RAGE signaling pathway in diabetic complications) as well as other cellular functions such as protein processing in endoplasmic reticulum, insulin resistance, and AHR (Figure 3A).

#### 3.1.2 Protein–Protein Interactions

Using the STRING database, we next aimed to identify protein–protein interactions between proteins coded by protein‐coding genes. Within the network of all interactions, 565 proteins with more than 20 interactions were identified, and the network of interactions is presented in Figure 3C. Proteins that showed >100 interactions and can be considered as proteins potentially affecting major cellular processes are presented in Figure 3B. Among these proteins are: serine/threonine kinase 1 (AKT1), glyceraldehyde‐3‐phosphate dehydrogenase (GAPDH), catenin beta 1 (CTNNB1), heat shock protein 90 α family class A member 1 (HSP90AA1), and eukaryotic translation elongation factor 2 (EEF2). To identify cellular functions of the highly interconnected proteins, we performed pathway enrichment analysis. The main pathways involved include the focal adhesion‐PI3K‐Akt‐mTOR‐signaling pathway; AhR; fluid shear stress and atherosclerosis; protein processing in endoplasmic reticulum; thermogenesis; adipogenesis; and several inflammatory‐related pathways (IL‐5, IL‐2, TGF‐β receptor, and IL‐6 signaling) (Figure 3C).

#### 3.1.3 Potential Regulators of Gene Expression by Transcription Factors

The next step of our bioinformatic analysis was to identify potential transcription factors whose regulatory activity could be modulated by OJ and be involved in the observed differential expression of protein‐coding genes, using the EnrichR tool. More than 38 potential transcription factors were identified (Table 1), including NFKB1, ATF4, PLAU, NRF1; YY1, RELA, specificity protein 1 (SP1), forkhead box O4 (FOXO4), promyelocytic leukemia protein (PML), AhR e AhR nuclear translocator (ARNT), peroxisome proliferator activated receptor alpha (PPAR‐α), and others (Table 1).

**TABLE 1:** Transcription factors potentially involved in the gene expression changes after 60 days of OJ consumption.

Transcription factor	Name	*p* value
NFKB1	Nuclear factor kappa B subunit 1	2.50E‐20
ATF4	Activating transcription factor 4	9.30E‐12
PLAU	Plasminogen activator, urokinase	3.10E‐11
CREM	cAMP responsive element modulator	5.60E‐08
NRF1	Nuclear respiratory factor 1	7.00E‐07
MYC	Proto‐oncogene	4.50E‐06
YY1	Yin‐Yang 1	9.70E‐06
ELK4	ETS transcription factor ELK4	2.80E‐05
RELA	Fator de transcrição p65	0.00016
RXRA	Retinoic acid receptor RXR‐alpha	0.00017
IRF9	Interferon regulatory factor 9	0.00023
TP53	Tumor protein 52 (TP53)	0.00024
SP1	Specificity protein 1	0.00037
XBP1	X‐box‐binding protein 1	0.0004
ESR1	Estrogen receptor alpha	0.00062
ARNT	Translocador nuclear do receptor de hidrocarboneto de arila	0.001
SMAD4	SMAD family member 4, mothers against decapentaplegic homolog 4	0.00131
TCF7L2	Transcription factor‐7‐like‐2	0.00246
CREB1	cAMP responsive element‐binding protein 1	0.00306
E2F1	E2F transcription factor 1	0.00317
MYB	Proto‐oncogene, T	0.00343
NFYA	Nuclear transcription factor Y subunit alpha	0.00398
CEBPD	CCAAT/enhancer‐binding protein delta	0.00632
NFIL3	Nuclear factor, interleukin 3	0.00815
REL	Proto‐oncogene c‐Rel	0.01004
FOXO4	Forkhead box O1	0.01112
STAT3	Signal transducer and activator of transcription 3	0.0128
SMAD3	Suppressor of mothers against decapentaplegic 3	0.01622
PPARA	Peroxisome proliferator‐activated receptor‐α	0.01853
EGR1	Early growth response factor 1	0.01867
EZH2	Enhancer of Zeste homolog 2	0.02143
KLF4	Kruppel‐like factor 4	0.02143
PML	Promyelocytic leukemia protein	0.02149
MEF2C	Myocyte enhancer factor 2 C	0.02723
PPARG	Peroxisome proliferator‐activated receptor gamma	0.02927
REPIN1	Replication initiator 1	0.03335
HIPK2	Homeodomain‐interacting protein kinase 2	0.03822
AHR	Aryl hydrocarbon receptor	0.03908

#### 3.1.4 Molecular Docking

**FIGURE 4:**
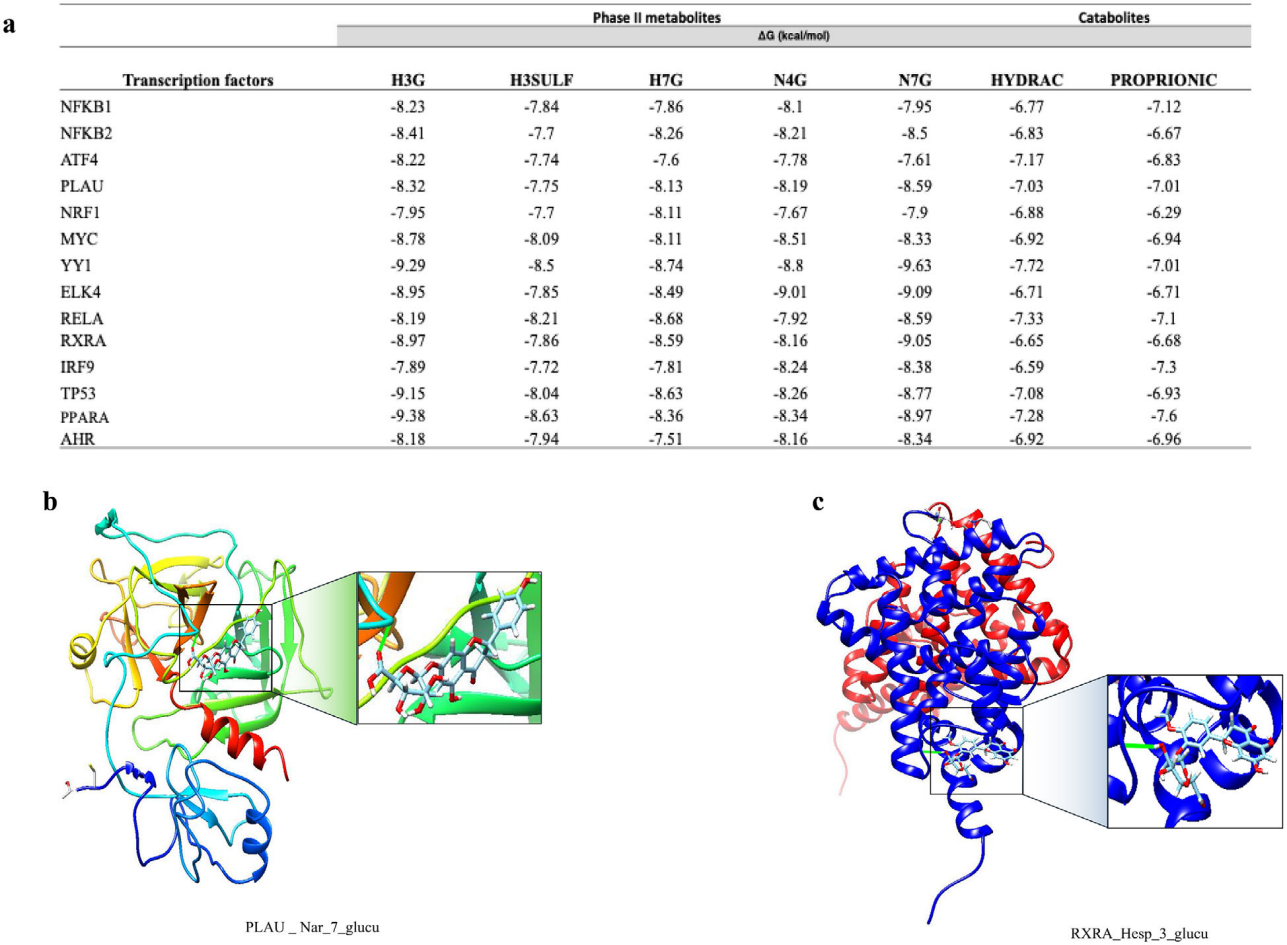
Molecular docking. (a) binding capacity of Phase II metabolites and catabolites with top transcription factors. (b) Figures present the strongest mode obtained by computational docking of Nar_7_glucu and plasminogen activator, urokinase (PLAU) transcription factor. (c) Figures present the strongest mode obtained by computational docking of Hesp_3_glucu and retinoic acid receptor RXR‐alpha (RXRA) transcription factor.

Following identification of potential transcription factors involved in the observed genomic changes, we aimed to determine whether flavanone Phase II metabolites and major citrus gut‐derived catabolites could exhibit binding affinities with these transcription factors using in‐silico 3D molecular docking. Molecular docking analysis revealed high potential binding between the metabolites and transcription factors, with free energy lower than −7 kcal/mol. The binding energy ranged from −6.29 to −9.63 kcal/mol. The highest binding was observed between Phase II flavanone metabolites and transcription factors, ranging from −7.6 to −9.63 kcal/mol (Figure 4B).

#### 3.1.5 Differentially Expressed Coding Genes Are Associated With Cardiometabolic and Nutrition Disorders

Next, we aimed to identify associations between the differentially expressed protein‐coding genes and known human diseases, using the Toxicogenomic Database, a tool that interrelates gene expression to diseases and can reveal potential roles in disease prevention or development. This analysis demonstrated that the differentially expressed genes are associated with heart diseases, vascular diseases, hypertension, diabetes, obesity, and others (Table 2).

**TABLE 2:** Associations of gene expression profile after 60 days of OJ consumption with known human diseases.

Disease name	*p* value
Cardiovascular diseases	
Heart diseases	1.26e‐30
Vascular diseases	1.55e‐25
Myocardial ischemia	5.51e‐24
Reperfusion injury	1.35e‐8
Brain ischemia	2.59e‐8
Cardiomyopathies	2.05e‐7
Myocardial infarction	3.07e‐7
Ventricular dysfunction	1.43e‐6
Hypertension	1.23e‐5
Stroke	2.63e‐5
Metabolic diseases	
Osteoporosis	4.06e‐11
Diabetes mellitus	8.91e‐11
Glucose metabolism disorders	1.00e‐10
Brain diseases, metabolic	4.76e‐8
Mitochondrial diseases	1.19e‐6
Nutrition disorders	
Obesity	9.76e‐9
Overweight	9.76e‐9

#### 3.1.6 Orange Juice Modulated Protein Noncoding RNAs

**FIGURE 5:**
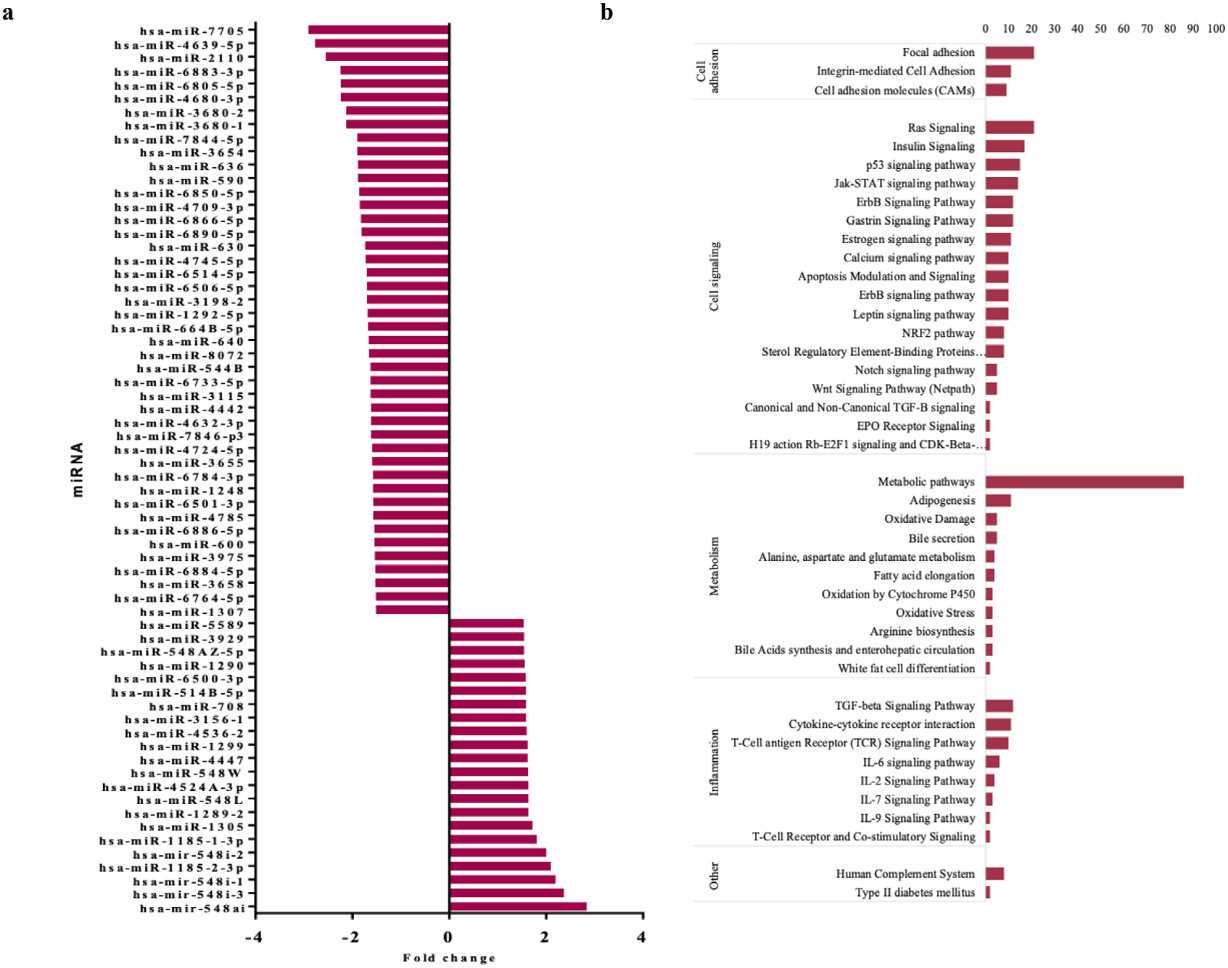
(a) miRNAs are differentially expressed in peripheral blood mononuclear cell (PBMC) after 60 days of orange juice (OJ) consumption. (b) Pathways in which the miRNA gene targets are involved.

The microarray analysis also allowed the identification of altered expression of noncoding RNAs after OJ consumption, including miRNAs. We observed significant modulation in the expression of 66 miRNAs, with 22 upregulated and 44 downregulated, with FC ranging from −1.52 to −2.91 for downregulated ones and from 1.54 to 2.84 for upregulated ones (Figure 5A). Following identification of miRNAs, we searched for potential target genes of these miRNAs, that is mRNAs to which miRNAs can bind and induce posttranscriptional modifications. We identified a total of 1286 potential target genes regulated by these miRNAs. Identification of miRNAs‐associated cellular functions was performed through pathway analysis of their target genes. These functions were grouped into four categories: cell adhesion, cell signaling, metabolism, and inflammation. Key pathways within these groups included Ras, insulin, and p53 signaling pathway, adipogenesis, oxidative damage, white fat cell differentiation, cytokine‐cytokine receptor interaction, and IL‐6 signaling pathway (Figure 5B).

**FIGURE 6:**
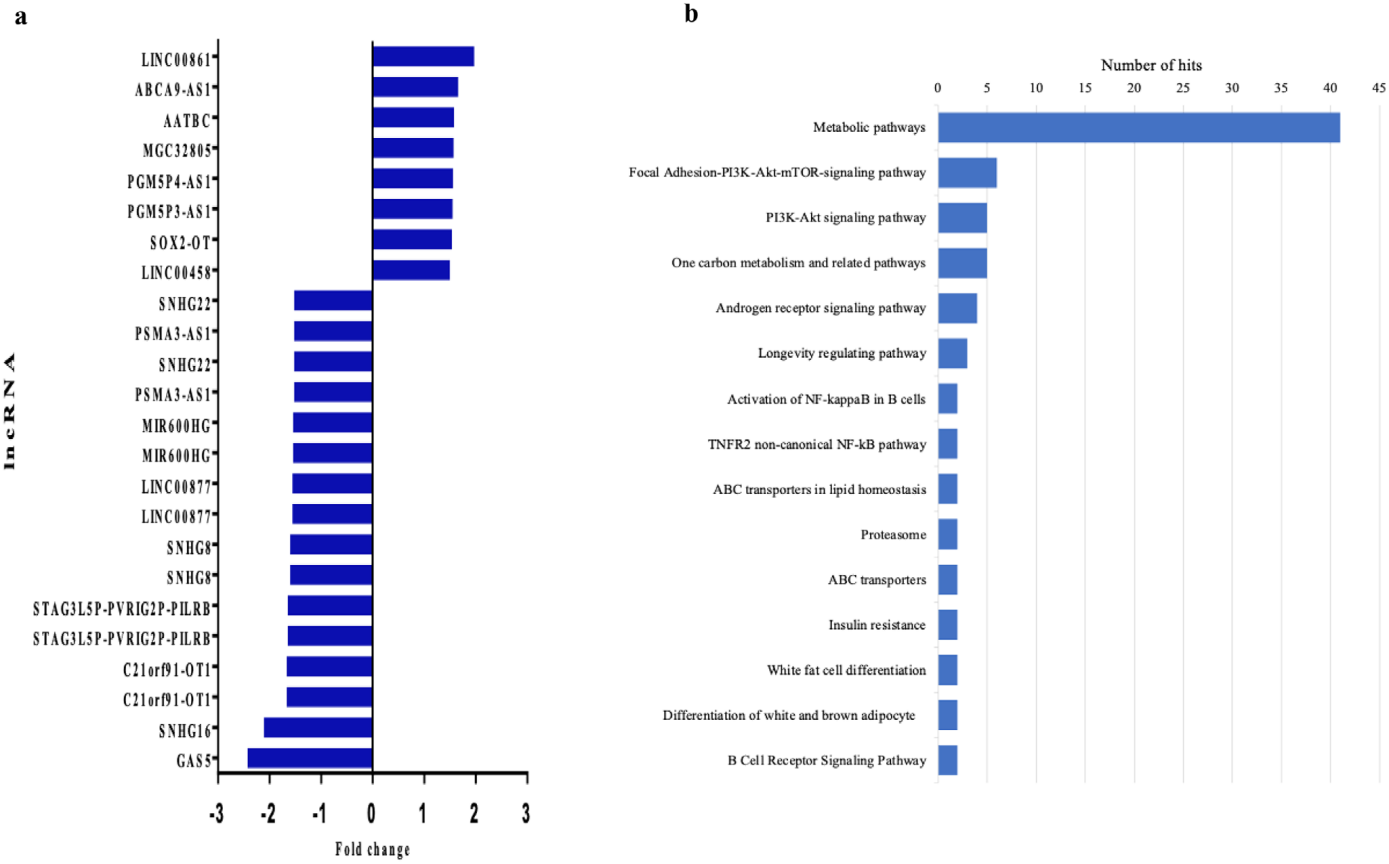
(a) lncRNA is differentially expressed in peripheral blood mononuclear cell (PBMC) after 60 days of orange juice (OJ) consumption. (b) Pathways in which the lncRNA gene targets are involved.

Moreover, OJ also modulated the expression of another group of non‐coding RNA, which are lncRNAs. A total of 19 lncRNA exhibited differential expression, with 11 downregulated and 8 upregulated (Figure 6A). Using the bioinformatic tool LncRRIsearch, we identified 642 potential target genes. Pathway enrichment analysis revealed that metabolic pathways had the highest number of hits, followed by main pathways such as focal adhesion‐PI3K‐Akt‐mTOR‐signaling, longevity regulating, NF‐kB signaling, ABC transporters involved lipid homeostasis, insulin resistance, white fat cell differentiation, and differentiation of white and brown adipocytes (Figure 6B).

Microarray analysis has also shown that OJ consumption can modulate the expression of another group of non‐coding RNA, the snoRNA. We observed a change in expression of 67 snoRNA differentially expressed, of which 6 were upregulated and 61 downregulated.

#### 3.1.7 Integrative Analysis

**FIGURE 7:**
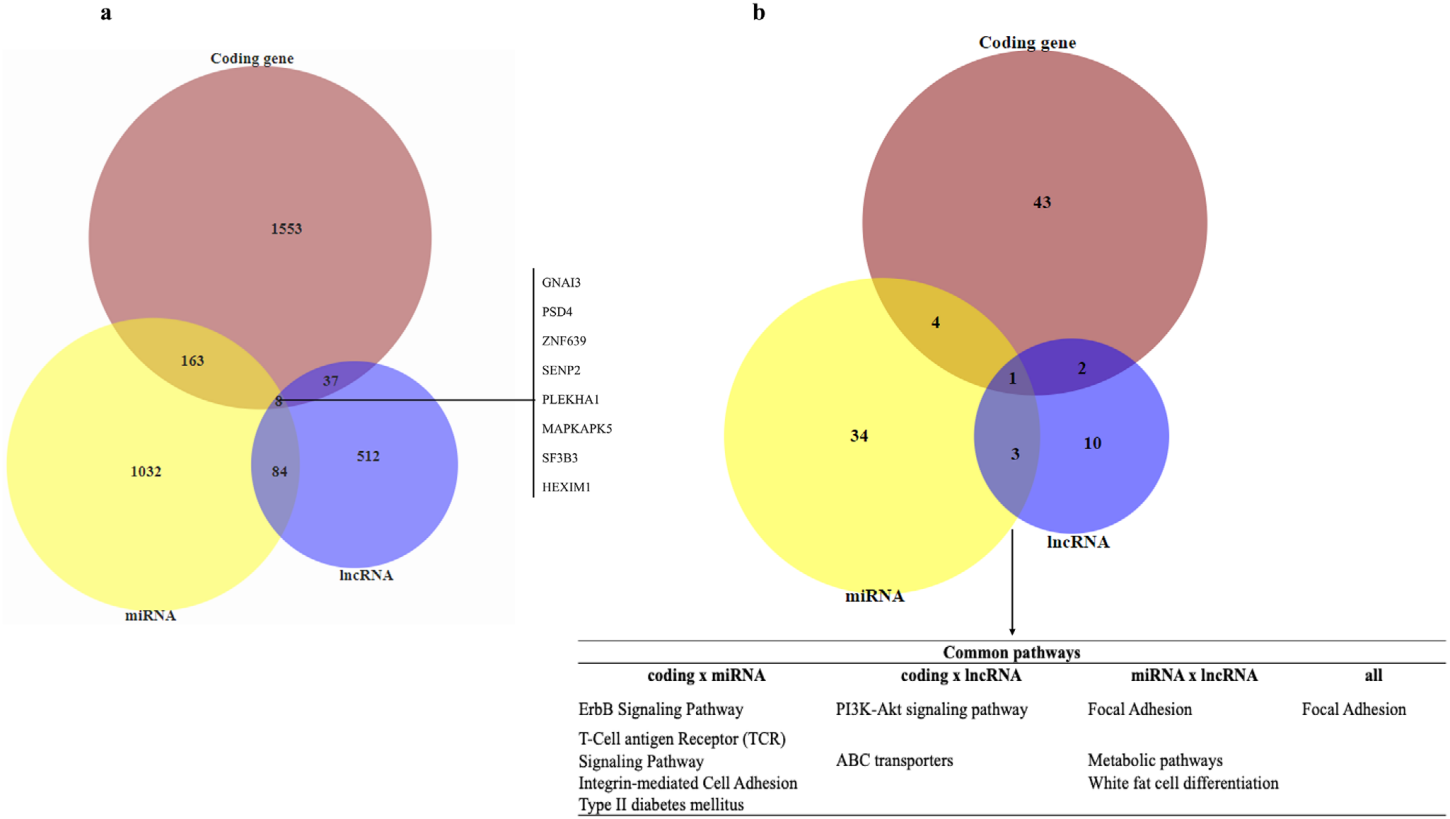
Data integration. (a) Venn diagram of common genes among coding genes, miRNA target genes, and long‐non‐coding RNA (lncRNA) target genes. (b) Venn diagram of common pathways among the coding genes, miRNA target genes, and lncRNA target genes.

Given the role of post‐transcriptional regulation through interactions among various classes of RNAs, we subsequently performed an integrative analysis to investigate the observed regulatory modulation. First, we compared the expression profile of differentially expressed coding genes, miRNA targets and lncRNA target genes (Figure 7A). This analysis identified eight genes in common among the groups (GNAI3, PSD4, ZNF639, SENP2, PLEKHA1, MAPKAPK5, SF3B3, and HEXIM1); 163 between coding genes and miRNA target genes (ELK4, CD55, NCBP3, GRB2, EIF4A3, MAPK1, and other); 87 between lncRNA and miRNA target genes (GABPB2, NXPE3, PGRMC2, CCL28, PLEC, BAG1, and other); and 37 between lncRNA and coding genes (ADAMTS4, AP1M1, AP1S3, ARHGEF39, B3GALNT2, C15orf40, and other) (Figure 7A).

We also compared the pathways shared among coding genes, lncRNA and miRNA gene target pathways. This analysis showed (Figure 7B): 1 pathway common to all groups (focal adhesion); 4 shared between coding genes and miRNA (ErbB signaling, T cell antigen receptor [TCR] signaling, integrin cell adhesion, and Type II diabetes mellitus); 2 between coding gene and lncRNA (PI3K‐Akt signaling and ABC transporters); and 3 shared between miRNA and lncRNA (focal adhesion, metabolic pathways, and white fat cell differentiation).

### 3.2 Consumption of Orange Juice Results in Differential Modulation of Global Gene Expression Profiles Between Overweight and Normal‐Weight Individuals

**FIGURE 8:**
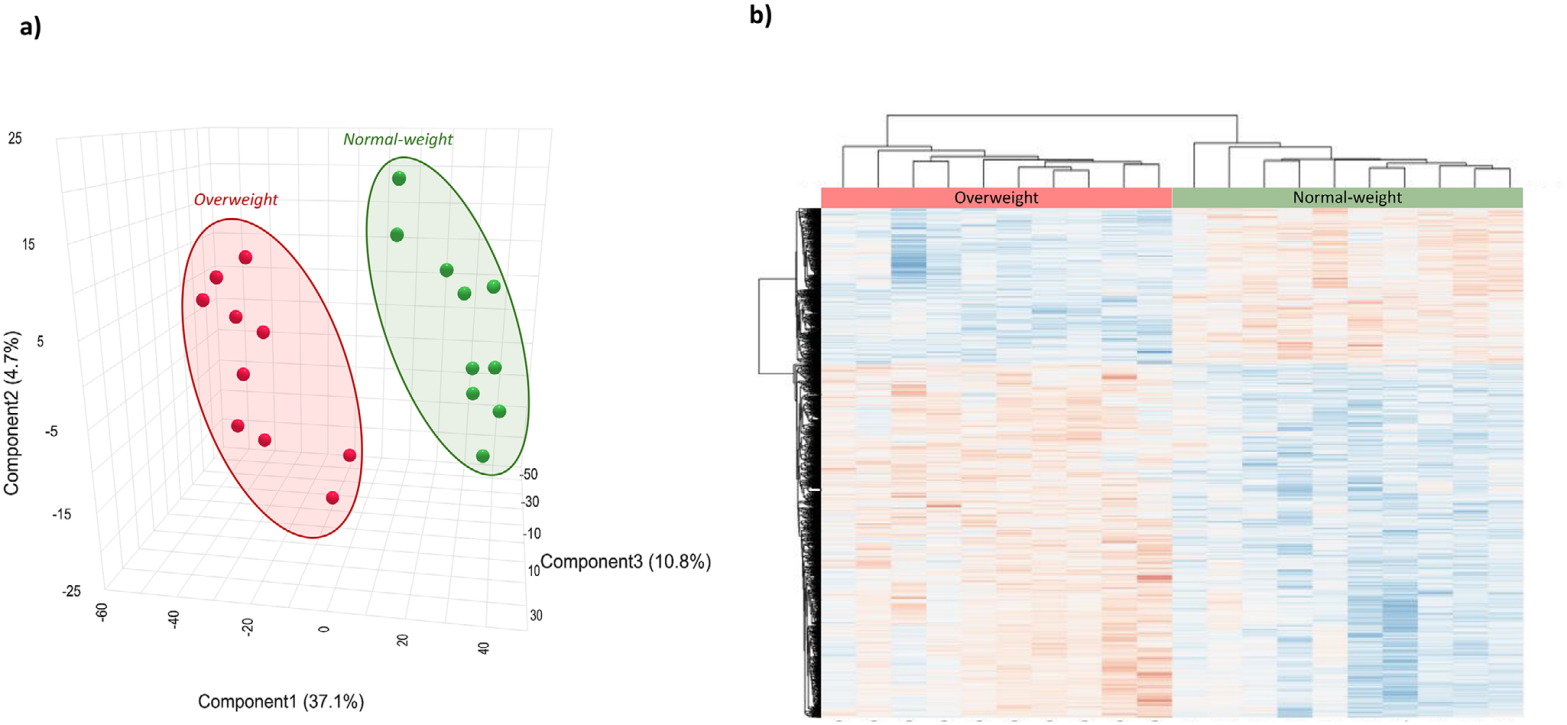
Comparison of global gene expression in peripheral blood mononuclear cells (PBMCs) of humans after orange juice consumption between normal‐weight (NW) and overweight (OW) volunteers. (a) Partial least squares‐discriminant analysis (PLS‐DA) of gene global expression profile between OW and NW groups. (b) Heatmap obtained with hierarchical cluster analysis of global gene expression between OW and NW groups.

To evaluate the potential effect of clinical parameters on nutrigenomic modifications in PBMCs induced by regular consumption of OJ, we considered subgroup analysis. In this study, we aimed to evaluate the impact of body weight on nutrigenomic modification by performing subgroup analyses in overweight (OW) and normal‐weight (NW) volunteers. Comparison of the global gene expression profile of OW and NW volunteers at 0 day did reveal slight differences between the two groups using PLSDA analysis (data not shown). For this raison, differentially expressed genes were calculated by comparing expression profile at Day 60–Day 0 for each of the two subgroups. First, we performed comparisons of global gene expression profiles applying PCA. As shown in Figure 8A, the gene expression profiles of OW and NW volunteers after 60 days of regular OJ consumption differ between them. We observed two distinct groups of profiles corresponding to the gene expression profiles of OW in 1 group and NW in another group. This observation suggests that regular OJ intake may impact gene expression differently according to BMI. Likewise, the hierarchical clustering of gene expression profiles shows two groups, one corresponding to OW volunteers and the second grouping the gene expression profiles of NW volunteers (Figure 8B) after 60 days of OJ consumption.

Following previous observations, our next step was to identify differentially expressed genes into population subgroups: OW and NW volunteers. In this way, 2741 oligonucleotides were identified as differentially expressed, among them 2222 were downregulated (fold change ranging from −15.6 to −1), and 519 were upregulated (fold change ranging from 1.5 to 6.72), in the OW group after OJ consumption, among which 1257 were protein‐coding genes, 63 were miRNA, 22 were lncRNA, and 95 were snoRNA. In the NW group, 2153 oligonucleotides differentially expressed were identified, among them 1519 were downregulated (fold change ranging from −17.95 to −1), and 634 were upregulated (fold change ranging from 1 to 166.85). We observed change in the expression of 1561 protein‐coding genes, among them 1479 being protein coding genes, 57 miRNA, 25 lncRNA, and 68 snoRNA.

#### 3.2.1 Comparative Analysis Between OW and NW Groups for Protein‐Coding Genes

**FIGURE 9:**
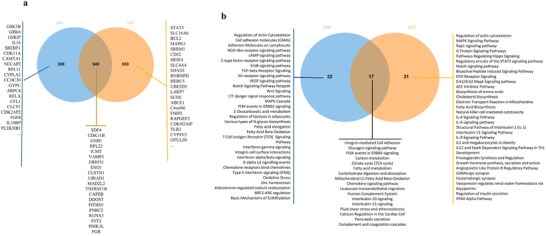
(a) Venn diagram of common coding genes between overweight (OW) and normal‐weight (NW) groups. (b) Venn diagram of common coding genes related pathways between OW and NW groups.

Considering protein‐coding genes, 949 were found to be common between the OW and NW groups, including SDF4, CDK11B, GNB1, VAMP3, ENO1, NAMPT, and others (Figure 9A). However, some genes were uniquely identified in each group. For example, GSK3B, GRK6, GSKIP, and IL16 were found only in the OW group, whereas STAT3, SLC16A6, BCL2, and MAPK1 were exclusive to the NW group.

We then performed pathway enrichment analysis for each group and compared significantly overrepresented. Seventeen pathways were common to both groups, including integrin‐mediated cell adhesion, glucagon signaling, PI3K signaling, fatty acid metabolism fatty acid beta‐oxidation, and others (Figure 9B). The cell adhesion molecules (CAMs), MAPK cascade, PI3K events in ERBB2 signaling, regulation of lipolysis in adipocytes, fatty acid elongation, and fatty acid beta oxidation pathway, were exclusively in the OW group. In contrast, the IL‐4, IL‐1, IL‐9 signaling, and interleukin‐11 signaling pathways were identified only in NW group (Figure 9B).

#### 3.2.2 Comparative Analysis Between OW and NW Groups for miRNA

**FIGURE 10:**
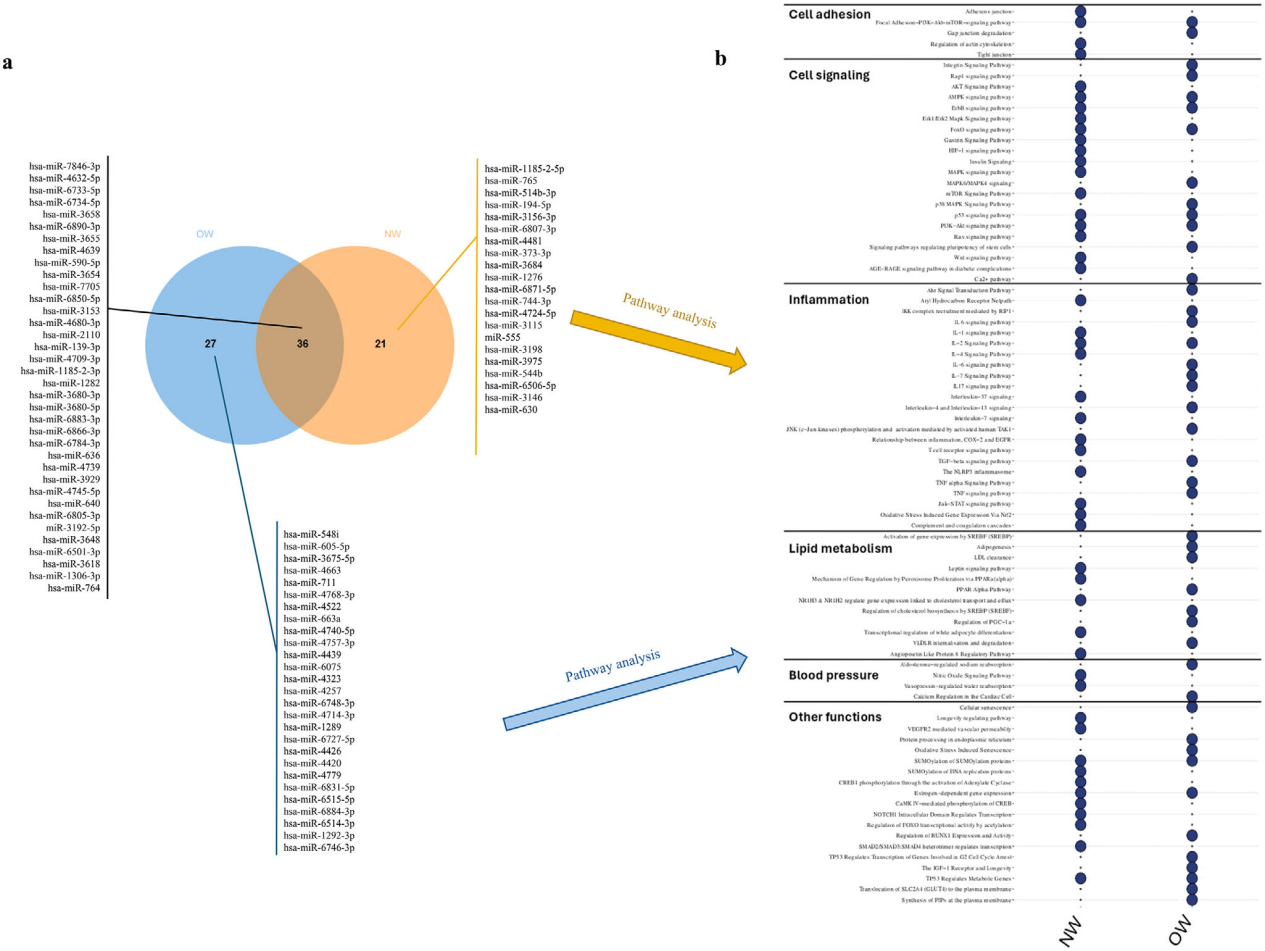
(a) Venn diagram of common miRNA differentially expressed after orange juice (OJ) consumption by overweight (OW) and normal‐weight (NW) groups. (b) Venn diagram of common pathways related to miRNA target genes by OW and NW groups.

We observed that 36 miRNAs were common between the groups, including miR‐7846‐3p, miR‐4632‐5p, miR‐6733‐5p, miR‐6734‐5p, miR‐3658, miR‐6890‐3p, miR‐3655, miR‐4639, miR‐590‐5p, miR‐3654, and others (Figure 10A). Following identification of potential targets of these miRNAs, we performed pathways analyses and comparative evaluation (Figure 10B). We observed that these genes are involved in several pathways, including the AMPK signaling pathway, estrogen‐dependent gene expression, focal adhesion‐PI3K‐Akt‐mTOR‐signaling pathway, FoxO signaling pathway translocation of SLC2A4 (GLUT4), and others. The miRNAs such as miR‐548i, miR‐1292‐3p, miR‐605‐5p, miR‐3675‐5p, and others (Figure 10B) were found to be differentially expressed only in the OW group, participating in adipogenesis, AhR and PPARα signaling, and the regulation of differentiation of white adipocytes.

In contrast, the miRNAs such as miR‐1185‐2‐5p, miR‐765, miR‐514b‐3p, miR‐194‐5p, and others were identified only in the NW group. These miRNAs are associated with pathways including the AKT signaling pathway, angiopoietin‐like protein 8 regulatory pathway, complement and coagulation cascades, and IL‐1 signaling pathway.

#### 3.2.3 Comparative Analysis Between OW and NW Groups for Associated Diseases

**FIGURE 11:**
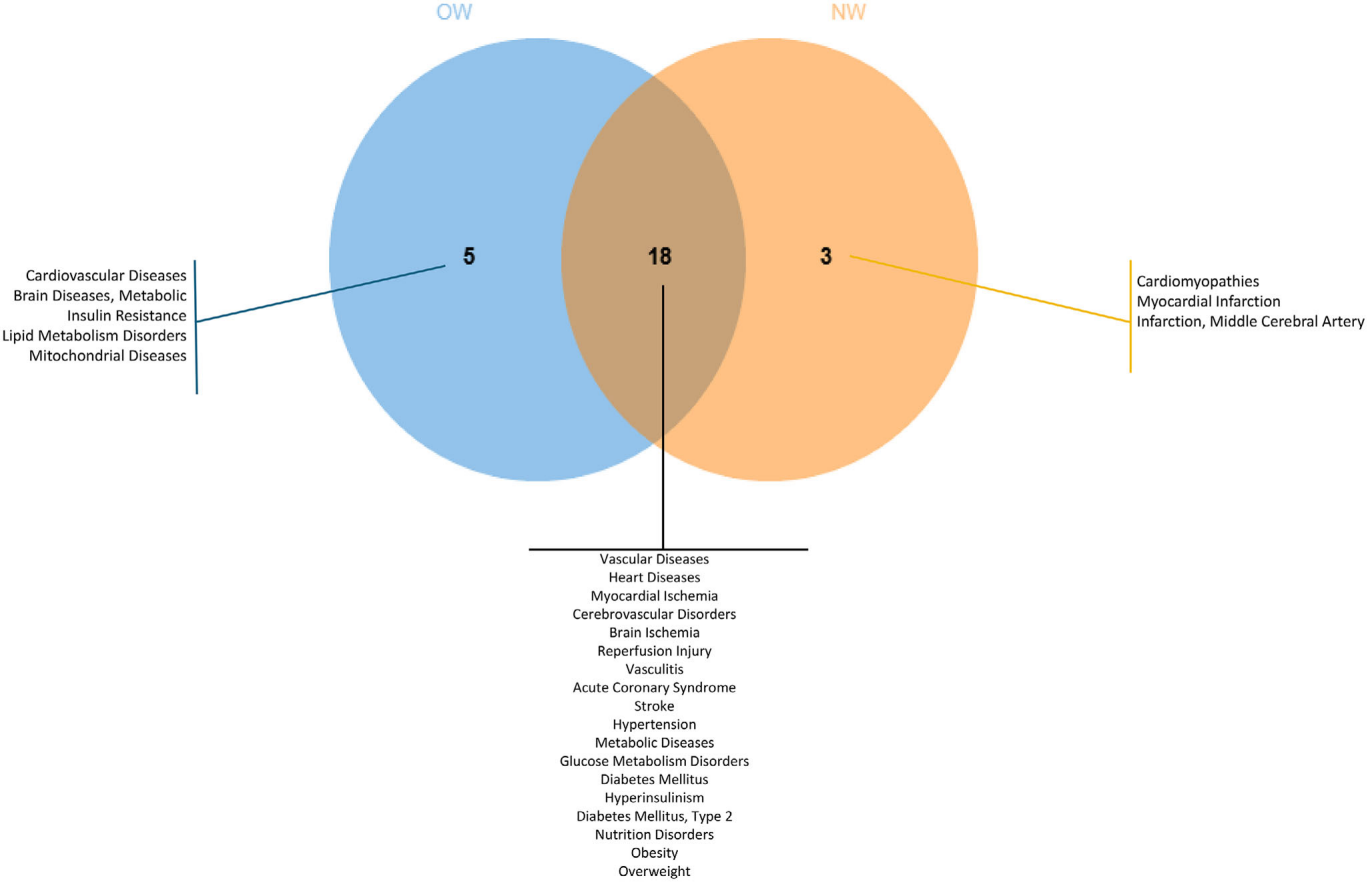
Venn diagram of common diseases associated with the differentially expressed genes for overweight (OW) and normal‐weight (NW) groups.

Using the Toxigenomic database tool, we also predicted diseases associated with the differentially expressed protein‐coding genes in both groups. Vascular disease, heart disease, hypertension, metabolic diseases, glucose metabolism, and obesity were associated with both the OW and NW groups. On the other hand, disease related to cardiovascular, brain, insulin resistance, lipid metabolism, and mitochondrial diseases were identified exclusively in the OW group. In contrast, cardiomyopathies, myocardial infarction, and middle cerebral artery diseases were associated with the NW group (Figure 11).

## 4 Discussion

OJ is the main dietary source of flavonoids for the Brazilian population [5, 21], and oranges are also the most widely consumed fruit worldwide [22], playing an important role in health promotion and in reducing the risk of developing chronic diseases [10, 11, 23, 24]. In this study, we explored the molecular mechanisms underlying the health benefits of chronic OJ consumption using a transcriptomic approach in PBMC.

We observed that chronic OJ consumption modulates the expression of coding and non‐coding RNAs (miRNA, lncRNA, and snoRNA) and identified pathways in which these genes are involved, primarily related to blood pressure regulation, inflammation, cell signaling, and metabolism pathways. Here, we also predicted transcription factors that may be modulated by chronic OJ consumption and potentially involved in the regulation of these genes. Although most findings were not reflected in biochemical and physiological parameters, some of them may contribute to the significant reductions in systolic and diastolic blood pressure and body fat percentage previously observed after 60 days of OJ consumption [1], supporting previous studies. Similarly, Milenkovic et al. [13] observed that the consumption of 500 mL of OJ for 4 weeks significantly affected the expression of 3422 genes in PBMCs. Among these, 1819 genes were modulated by hesperidin and were involved in processes such as chemotaxis, adhesion, infiltration, and lipid transport. Likewise, the flavanone metabolites, hesperetin 3′‐sulfate, hesperetin 3′‐glucuronide, and naringenin 4′‐glucuronide, modulate the expression of genes related to inflammation, atherogenesis, cell adhesion, and cytoskeletal organization [25]. Furthermore, Pla‐Pagà et al. [14] reported changes in global gene expression of specific coding genes related to blood pressure regulation and inflammation after consumption of 500 mL/day of OJ for 12 weeks. It is important to highlight that these previous studies evaluated only global protein‐gene expression. In contrast, we observed differential expression in both coding and non‐coding genes, providing a more integrative and comprehensive view of gene expression regulation following the intervention.

In this context, among the pathways involved blood pressure regulation, we identified the aldosterone synthesis and secretion, renin secretion, ACE inhibitor, and other, as being affected by OJ consumption. Genes such as NAMPT (−7.37) and NLRP3 (−2.86), which are found upregulated in hypertensive individuals, showed reduced expression. The downregulation in expression of this gene is associated with a positive effect on blood pressure reduction [14, 15]. These data support a previous study reporting reduced blood pressure in prehypertensive and mildly hypertensive individuals, along with decreased NAMPT expression, following 12 weeks of daily consumption of 500 mL of OJ [14]. In addition to these genes, for the first time, we demonstrated that NR4A2 (−23.74), PER1 (−2.25), SIK1 (−1.94), GPR183 (−3.86), and SGK1 (−4.35) genes were downregulated after OJ consumption. These genes have been shown to be upregulated in hypertensive individuals and downregulated in normotensive ones [26]. The downregulation of SGK1 gene (−4.35) by OJ is particularly relevant, as SGK1 deficiency has been associated to the prevention of increased blood pressure [27]. Furthermore, activity of the SIK1 is elevated in proximal tubule cells of hypertensive rats, and its silencing modulates active sodium transport [28]. The NR4A2 gene has been reported to be important for cytokine regulation and may contribute to inflammation in the renal medulla in hypertension; the activation of NR4A2 in response to angiotensin II is further modulated by SIK1 [26]. Another gene identified here as downregulated was GPR183 (−3.86). Although poorly explored, previous studies have shown that GPR183 expression was increased in mice with hypertension, and renal biopsies from individuals with hypertensive nephropathy have demonstrated the deficiency of this gene in the endothelium. This suggests a cardioprotective effect after OJ consumption [29] by downregulation of this gene. Therefore, OJ consumption may modulate several genes related to blood pressure homeostasis, representing one of potential mechanism through which OJ contributes to the blood pressure reduction observed in pre‐hypertensive individuals [14], as well as in eutrophic and OW [11], and Classes II and III obese individuals [23].

The inflammatory‐related genes were also modulated by the OJ consumption. The TCR signaling pathway, which mediates the activation of NF‐kB, was one of the pathways affected [30]. The NF‐κB pathway plays a crucial role in regulating inflammatory responses to stress, and its activation is directly associated with increased inflammation, oxidative stress. It is commonly overexpressed in conditions such as chronic inflammation [31], obesity [32], and diabetes [33]. In our study, several genes involved in this signaling pathway were downregulated following the intervention, including NFKBIZ (−3.98), IL1B (−72), IL6 (−2), PTGS2 (−6.13), and RGS1 (−30.3). Previous studies have shown that FKBIZ gene expression is induced following activation of NF‐κB, which in turn induces proinflammatory cytokines such as IL‐6 [34]. A similar pattern has been observed for the PTGS2 gene [35]. Regarding GS1 gene, silencing this gene has been reported to attenuate inflammation by reducing activation of the NF‐κB and p38 axis, thereby reducing the synthesis of cytokines IL‐1β and IL‐6 [36]. These findings are consistent with previous in vitro and in vivo studies demonstrating that hesperetin improves inflammation in white adipose tissue by reducing NF‐κB activation, and the expression of IL‐1β, IL‐6, and the macrophage activation marker PTGS2 [37]. Supporting these findings, it was previously observed that PML was upregulated by OJ consumption, with a 28.2‐fold increase [38]. This protein has been reported to interact with the inflammasome to reduce inflammation, leading to decrease levels of proinflammatory interleukins such as IL‐1β [39]. Taken together, the expression profile of these genes following the consumption of OJ is suggestive of antiinflammatory effect of this fruit.

Bioinformatic analyses also revealed that differentially expressed genes following OJ intake are involved in the PI3K‐Akt signaling pathway, whose dysregulation has been associated with the diseases such as obesity and diabetes [40]. The PI3K pathway participates in the recruitment of inflammatory cells, and PI3K signaling plays a fundamental role in lipid and glucose metabolism [41]. Kobayashi et al. [42] demonstrated that inhibition of PI3Kγ improved obesity‐induced insulin resistance by reducing macrophage infiltration and downregulating proinflammatory cytokines such as IL‐6 and IL‐1β. Additionally, genes involved in white fat differentiation and adipogenesis pathways were downregulated by OJ consumption, including KLF4 (−2.3), RIPK1 (−1.7), PLIN2 (−1.99), and CXCL8 (−34.43); genes found as overexpressed in obesity [43, 44, 45, 46]. Taken together, these findings strongly suggest that OJ may attenuate inflammation and modulate lipid metabolism through the regulation of key genes involved in these pathways.

Beyond coding RNAs, OJ consumption also modulates non‐coding RNAs, including miRNA, lncRNA, and snoRNA. In our study, miR‐548 (2.84) and miR‐1185‐1 (2.1) were found to be upregulated. These miRNAs have previously been found to be overexpressed and identified as biomarkers of response to weight‐loss diets. One proposed mechanism suggests that they may contribute to the negative regulation of the proinflammatory gene GSK3B [47], which was downregulated in our study (−1.52). Additionally, we observed significant downregulation of miR‐640 (−1.67) and miR‐1248 (−1.58), both known to be overexpressed during an inflammatory process [48]. Conversely, miR‐1305 (1.72) was upregulated, a change associated with attenuation of inflammation [49, 50].

Together with miRNAs, we also observed that OJ also modulates other types of non‐coding RNAs. The role of lncRNA and snoRNA in gene regulation remains poorly understood, as information on these non‐coding RNAs is still limited. However, emerging evidence suggests their involvement in key regulatory mechanism of gene expression. Among these, lncRNA SNHG16, which was downregulated in our study (−2.11), has been linked to diabetes‐related complications and vascular dysfunction [51]. lncRNA SNHG16 has been reported to be overexpressed in the serum of diabetic patients, through upregulation of Krüppel‐like factor 9 (KLF9) [52], a transcription factor that we found to be downregulated in our study (−1.71). Moreover, the lncRNA INC00861 (1.97) was positively regulated in our study; its downregulation has previously been associated with obesity [53]. We also observed upregulation of expression of lncRNA, AATBC (1.58), a human‐specific regulator of adipocyte plasticity that has been found to be overexpressed under thermogenic conditions. AATBC is currently considered a novel regulator of adipocyte plasticity and mitochondrial function in humans, with a potential link to obesity [54]. Furthermore, regarding snoRNAs, the RpL13a snoRNAs (SNORD U32, U33, U34, and U35) were downregulated (−1.97) following OJ intervention. A previous study has shown that these snoRNAs accumulate in the cytoplasm under lipotoxic or oxidative stress conditions [55]. Nevertheless, a reduction in of RpL13a snoRNAs expression has been associated with improved mitochondrial metabolism, decreased reactive oxygen species levels [56], and reduced the inflammation [57].

Bioinformatic analysis suggests that OJ consumption likely modulates several transcription factors involved in gene expression changes. Our prediction indicates that some of these transcription factors include NFKB, RELA, PLAU, NRF1, AHR, PPARA, and SP1, among others. This highlights OJ's potential influence on key biological processes such as inflammation, lipid metabolism, glucose metabolism, oxidative stress, and hypertension [33, 58, 59, 60]. Notably, this study provides unprecedented data contributing to a better understanding of the molecular mechanisms of OJ consumption over 60 days, analyzing both coding and non‐coding genes, while considering individual characteristics. We also performed subgroup analyses based on body mass index, considering NW and OW groups, which allowed us to evaluate BMI's impact BMI on nutrigenomic responses. Few studies have explored these differences based in gene expression in human cells, as we did here.

The analysis allowed us to observe not only what these groups have in common but also what makes them unique in their molecular response to chronic OJ consumption. Although some pathways were similarly modulated in both groups such as PI3K signaling and integrin‐mediated cell adhesion specific changes were observed in each group. In the OW group, modulation was observed mainly in lipid metabolism pathways, including regulation of lipolysis in adipocytes, fatty acid elongation, and beta‐oxidation, which were only identified in this group. We also identified exclusive regulation of genes involved in these pathways, such as GSK3B and GRK6, and miRNAs miR‐548i and miR‐1292‐3p, previously linked to body weight control [47]. In the NW group, we observed modulation mainly of inflammation‐related pathways, such as IL‐4 and IL‐1 signaling. Genes like STAT3 and BCL2 were uniquely modulated. These results highlight how BMI can influence the body's response to OJ consumption and underscore the importance of considering these differences in nutrition studies. Additionally, our results reinforce the concept that individuals respond differently to polyphenol‐rich foods. This interindividual variability has been associated with genetics, gut microbiota, sex, and metabolic state [61, 62, 63].

## 5 Conclusion

Our study reinforces the therapeutic potential of OJ by providing important and unprecedented insights into the molecular mechanisms behind the effects of chronic OJ consumption. It suggests that it may improve blood pressure regulation, lipid metabolism, and inflammation, among other processes, by modulating gene expression, thereby contributing to cardiovascular health benefits. Moreover, we demonstrated that these nutrigenomic effects can vary according to individuals’ body weight. These findings offer valuable information for personalized dietary recommendations regarding the consumption of flavonoid‐rich foods.

## Funding

This work was funded by the Food Research Center (FoRC); the Research, Innovation and Dissemination Center (CEPID) of the São Paulo Research Foundation (FAPESP), São Paulo, State of São Paulo, Brazil [grant number 2013/07914‐8]; São Paulo Research Foundation (FAPESP), São Paulo, State of São Paulo, Brazil [grant numbers 2022/05463‐8 and 2024/03926‐6]; and the National Council for Scientific and Technological Development (CNPq) Grants 141878/2019‐3 and 314894/2021‐7) by CAPES (Grant 88887.512050/2020‐00). DM was supported by the United States Department of Agriculture, National Institute of Food and Agriculture (USDA‐NIFA), Hatch project 7010153.

## Conflicts of Interest

The authors declare no conflicts of interest.

## Data Availability

The data that support the findings of this study are available from the corresponding author upon reasonable request.
